# Origin of Low-Lying Red States in the Lhca4 Light-Harvesting Complex of Photosystem I

**DOI:** 10.1021/acs.jpclett.3c02091

**Published:** 2023-09-13

**Authors:** Vladislav Sláma, Lorenzo Cupellini, Vincenzo Mascoli, Nicoletta Liguori, Roberta Croce, Benedetta Mennucci

**Affiliations:** † Department of Chemistry and Industrial Chemistry, University of Pisa, 56124 Pisa, Italy; ‡ Department of Physics and Astronomy, Faculty of Science, Vrije Universiteit Amsterdam, 1082 HV Amsterdam, Netherlands

## Abstract

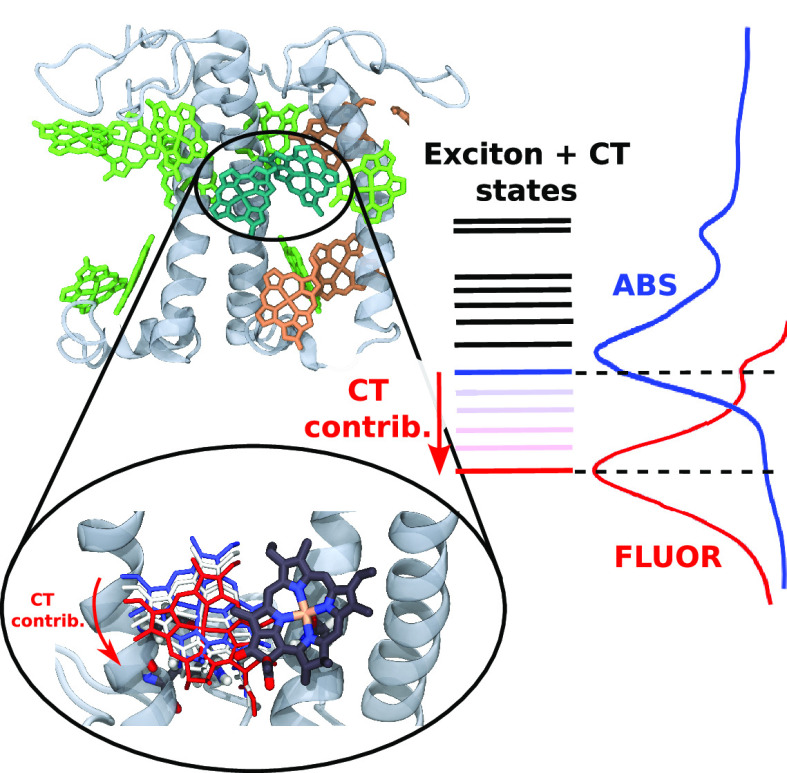

The antenna complexes of Photosystem I present low-lying states visible as red-shifted and broadened absorption and fluorescence bands. Among these, Lhca4 has the most evident features of these “red” states, with a fluorescence band shifted by more than 25 nm from typical LHC emission. This signal arises from a mixing of exciton and charge-transfer (CT) states within the excitonically coupled a603–a609 chlorophyll (Chl) dimer. Here we combine molecular dynamics, multiscale quantum chemical calculations, and spectral simulations to uncover the molecular mechanism for the formation and tuning of exciton-CT interactions in Lhca4. We show that the coupling between exciton and CT states is extremely sensitive to tiny variations in the Chl dimer arrangement, explaining both the red-shifted bands and the switch between conformations with blue and red emission observed in single-molecule spectroscopy. Finally, we show that mutating the axial ligand of a603 diminishes the exciton–CT coupling, removing any red-state fingerprint.

Photosystem I (PSI) is a multiprotein complex located in the thylakoid membrane of higher plants, algae, and cyanobacteria, playing a pivotal role in the initial stages of oxygenic photosynthesis by reducing ferredoxin and oxidizing plastocyanin.^1−3^ In plants and algae, PSI is composed of two parts: the core complex, where charge separation occurs, and the outer antenna complexes, known as Light-Harvesting Complex I (LHCI). The primary function of LHCI is to increase the absorption cross section of PSI, by harvesting light and transferring the excitation energy to the reaction center. The plant LHCI antenna is composed of four subunits, the products of the *Lhca1-4* genes. The corresponding proteins exhibit the typical structure found in the members of the light-harvesting complex multigenic family, characterized by three transmembrane helices spanning the thylakoid membrane.^4^ Each protein coordinates 13–15 Chlorophylls (Chls a and b) and 3–4 carotenoids within highly conserved binding sites.^5,6^ A key characteristic of the LHCI complexes is the presence of low-lying red states, also called “red forms”, that absorb light at wavelengths longer than the reaction center, P700.^2^ These states exhibit broad bandwidths, even at 4 K, extending the absorption of the complex to the far red and yielding exceptionally red-shifted emission. However, they also slow down the transfer of excitation energy to the reaction center, as energy migration needs to be thermally activated.^7−9^ Furthermore, the fluorescence of the Lhcas presents two emission components with different spectra and lifetimes, indicating the existence of at least two stable conformations of each complex.^10,11^ Switching between such conformations was also observed in single molecule fluorescence experiments.^12^

Mutation analysis of the Lhcas allowed determining the origin of the red states, which was attributed to the interplay of excitonically coupled states and CT states of chlorophylls a603 and a609.^13−15^ It was found that even a single mutation at the ligand for Chl a603, N98H, resulted in the loss of the low-lying red states and the red-shifted fluorescence.^13^ While the origin of the low-lying red states in LHCI complexes is known, the precise mechanisms of the formation of these states and the factors influencing their properties remain elusive. This is particularly interesting since the structures of many members of the LHC family are now available^4^ and they all show very small differences in the binding sites of Chl 603 and 609, indicating that minimal differences in the protein structure have a large effect on the spectroscopic properties of the pigments associated with it.

In this study, we undertook a theoretical investigation into the formation of low-lying red states in the light-harvesting antennas, focusing on Lhca4, which exhibits the largest red shift among all LHCI complexes.^16−18^ We combined molecular dynamics simulation to sample the Lhca4 conformational space with multiscale quantum mechanical calculations using the QM/MMpol approach,^19^ along with spectral modeling to investigate structural properties leading to the formation of low-lying states. The modeling is validated against experimental optical spectra of the wild type (WT) complex and mutant Lhca4-N98H, in which no low-lying red states are observed. From our calculations, we not only explain the strong dependence of the low-lying red states on geometrical properties of the Chl a603–a609 dimer and predict the effect of the mutation but also elucidate the origin of the two emission bands observed in the steady state fluorescence spectra.

We performed 4 MD replicas (MD1-MD4) for Lhca4 WT embedded in a lipid membrane and solvent (Figure 1), as well as a simulation of the N98H mutant. For the WT, we directly used the available crystal structure by Mazor et al.^6^ as a starting point for the simulation. The N98H mutant was first equilibrated through a united-atom simulation, allowing a faster relaxation of the system following the point mutation, and then sampled using the same protocol as for the wild type (see Section S2 in the Supporting Information). The structures of the Chl a603 and a609 binding sites for the WT and the mutant are visualized in Figure 1c-d. Three of the four replicas of the WT showed a very similar arrangement of the a603–a609 pair. However, the fourth replica was different in that a water molecule entered between Chl a603 and its ligand, Asn98, forming a stable hydrogen bond with the oxygen atom of Asn98 and binding the Mg atom of the chlorophyll (Figures S1 and S2). The bridging water in the a603 binding site was not observed in the other replicas. For this reason and the sake of simplicity, we regard this change as a rare event, and discuss this replica separately.

**Figure 1:**
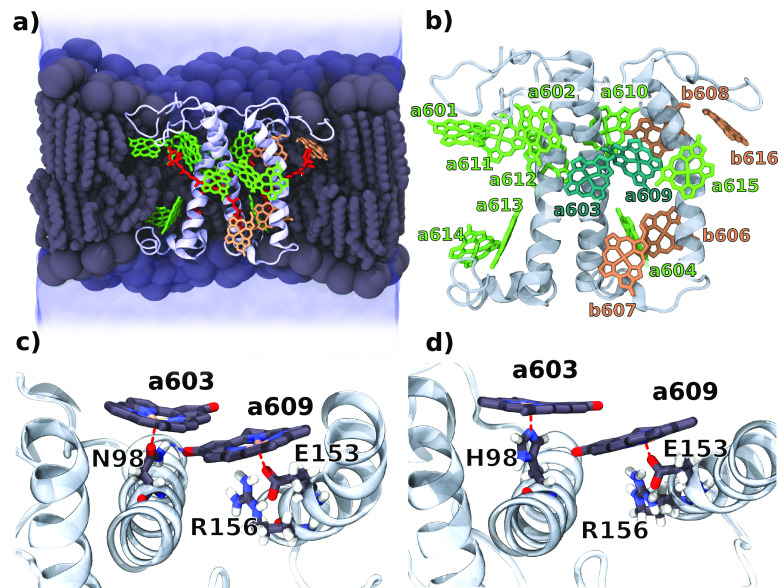
Molecular structure of Lhca4. (a) Longitudinal section of the simulated system. The Lhca4 complex was embedded in the phospholipid bilayer (gray) and water solution (blue). Pigment composition and protein orientation in the membrane are highlighted in the picture. Chls a are represented in green, Chls b in orange, and carotenoids in red. (b) Chl positions on the Lhca4 complex. The dimer a603–a609 is highlighted. (c) Binding pocket of Chls a603–a609 in the WT with highlighted binding residues. (d) Binding pocket of Chls a603–a609 in N98H. The red dashed lines represent coordination to the Mg atom, while the blue dashed line represents the hydrogen bond between Asn and Chl a609.

All three replicas of the WT (MD1–3) show similar structures of the protein backbone, which can be visualized on the 2D RMSD plots (Figure S3 in the SI). The largest differences are observed for the lumenal and stromal loops, which are clearly more mobile than the transmembrane helices, and for helix D. For a few frames of MD2, helix D is slightly rotated in the plane defined by the membrane relative to all the other conformations of the WT structure. In general MD2 and MD3 show more similarity among them than with MD1. Much larger differences are instead observed for the N98H mutant, especially for helix C, which is shifted toward the stromal side of the membrane (Figure S4 in the SI). Helix C contains the ligand of Chl a609; hence these changes might have a significant effect on the CT state formed between Chl a603 and a609.

The center-to-center a603–a609 distance is consistent among the replicas and around 0.7 Å larger than observed in the crystal structure, 8.82 Å for the crystal and 9.55 for the MD structures, respectively (Figure S5 in the SI). The distribution of this distance is significantly shifted to higher values for the N98H mutant. This is a clear effect of the His binding residue, which has a larger volume than the Asn present in the WT. Even though this shift is quite small (0.2 Å on average), its effect on the CT properties might be significant, given the sensitivity of CT couplings to the distance.^20^ The mutual orientation between the chlorophylls is conserved among the WT replicas and with the mutant, leading to a well-defined and consistent orientation of the Chl pair. The small variations in the relative positions and orientations of the Chls between the replicas correspond essentially to their mutual sliding in the plane defined by the conjugation ring.

The most significant difference between the WT and the mutant is the distance between the Chl a603 central Mg and the ligand, namely, Asn98 in the WT and His98 in the N98H mutant (Figure S5 in the SI). The broader distribution of the binding distances for the WT suggests weaker binding of the Chl by Asn, whereas the very narrow distribution in the mutant reflects the strong interaction of the Chl with His, which is the main Chl binding residue in LH systems.^5,6,21,22^ Unlike Chl a603, the binding of Chl a609 to Glu153 is strong and consistent among the replicas and does not change in the mutant. The weaker binding energy between Chl and Asn seems to be the reason for the insertion of a water molecule bridging the Asn98–Mg interaction in MD4.

In WT Lhca4, the Asn98 amide group also forms a hydrogen bond with the carbonyl group of Chl a609 (Figure 1c), which is maintained through the MD simulation for all of the replicas (Figure S5), although it is slightly stronger in MD1. This hydrogen bond stabilizes a closely packed arrangement of Chl a609 (Figure S6 in the SI). Clearly, this interaction is not present when Asn is replaced by His, as the His N–H group does not have the correct orientation to make the H-bond. As a result, not only the mutual distance between Chls is increased due to the different size of the ligand, but also the chlorophylls are not kept in the same tight conformation due to the missing hydrogen bond. Although these changes are quite small, their combined contribution might have a substantial effect on the properties of the CT state formed within this Chl pair.

In order to investigate the factors influencing the CT properties, we extracted 30 and 36 structures from MD1 and MD2, respectively, and performed QM/MMpol calculations^19,23^ of the CT states (see Section S3 in the Supporting Information). The CT properties are very sensitive to the structure of the CT dimer and the surrounding environment, especially if nearby charged residues are present. Therefore, prior to QM/MMPol calculations, we optimized the a603–a609 pair and its binding pocket at the QM/MM level (see Section S3 in the SI). This allows us to overcome the possible biases due to the empirical force field while allowing an exhaustive sampling of the conformations from MD.

In order to identify an effective intermolecular coordinate that can be linked to the CT properties, we considered a linear combination of all intermolecular atom–atom distances within the π-conjugated rings of the a603–a609 pair. The coefficients of the linear combination were obtained from a principal component analysis (PCA) of the intermolecular distances, as the first principal component represents the combination with maximum variability among the selected frames. Visually, this coordinate can be represented as a mutual sliding of chlorophylls a603 and a609 in the plane defined by the π-conjugated ring, as shown in Figure 2a. Importantly, this mutual sliding between Chls does not significantly change the center-to-center distance, but it substantially impacts the molecular orbital (MO) overlap. For negative values of the coordinate, the Chls of the CT pair are more aligned and have a larger contact surface; increasing the values of the coordinate instead lowers the overlap. The MO overlap is directly connected to the couplings between locally excited (LE) and CT states,^24^ which explains the variation of these couplings with the intermolecular coordinate (Figure 2c). Lowering the coordinate to even more negative values would eventually lead back to a lower MO overlap and thus a lower coupling between LE and CT states.

**Figure 2:**
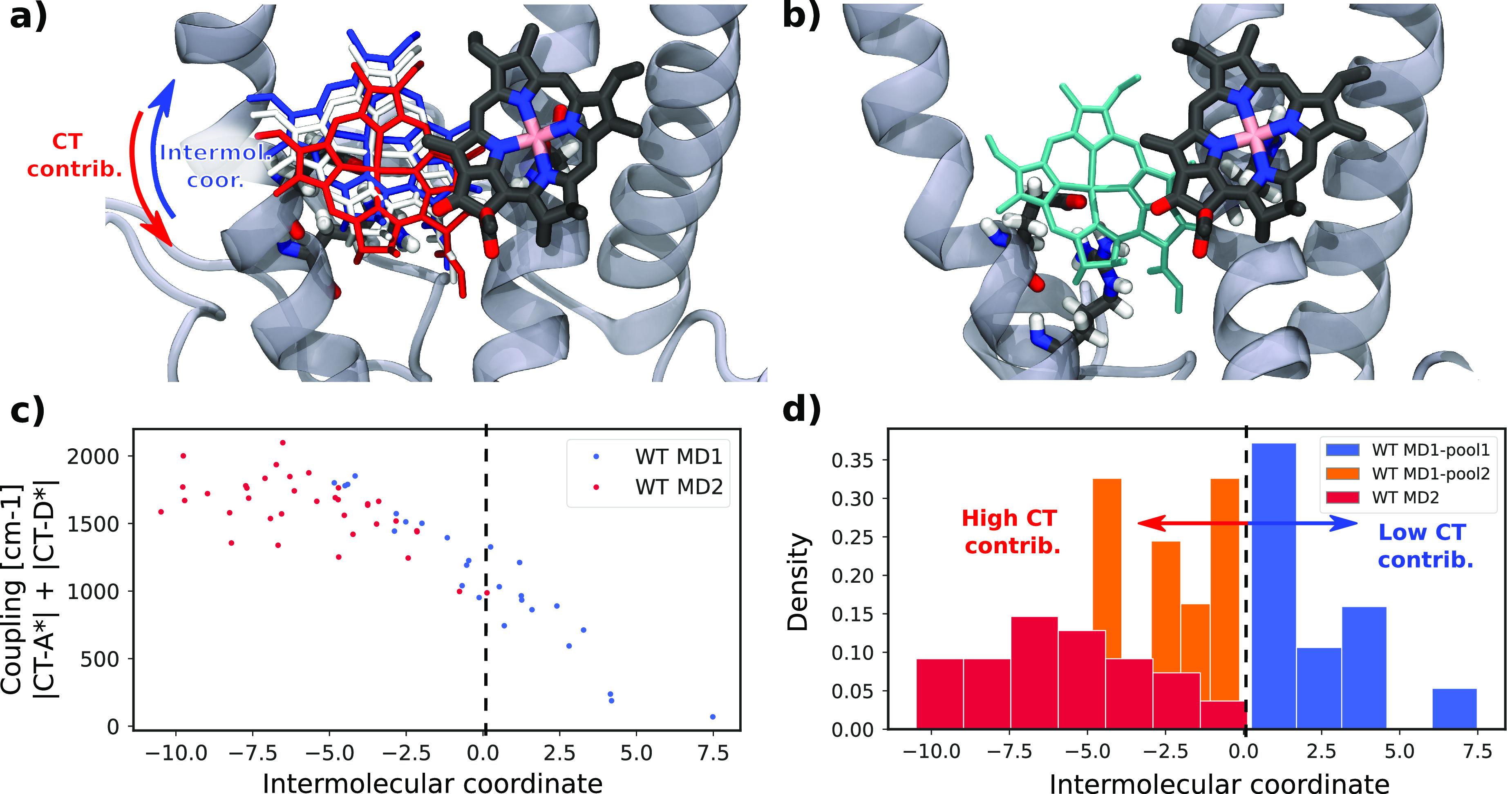
Geometrical analysis of the CT pair. (a) Structural representation of the intermolecular coordinate obtained by principal component analysis of the interatomic distances. Red (blue) structures represent lower (higher) values of the coordinate. (b) CT pair for the N98H mutant shown with the same orientation of the Chl a603 as for the WT. (c) Dependence of the coupling between LE and CT states on the intermolecular coordinate. (d) Projection of all the WT structures into the intermolecular coordinate and separation of the structures from the first replica (MD1) according to the similarity with the second replica (MD2).

Based on the sign of this intermolecular coordinate, we categorized the structures into different pools. Notably, the configurations of MD1 divide evenly into positive and negative values of the coordinate (and we collect them in MD1-pool1 and MD1-pool2, respectively), while MD2 frames fall exclusively in the negative region with substantial MO overlap (Figure 2d). In the N98H mutant, the CT pair is in a significantly different configuration due to the binding pocket and different binding residue (Figure 2b and Figure 1d). Calculating the intermolecular coordinate for this pair leads to very large values, indicating the specificity of the mutant with respect to all the WT structures. We thus assigned N98H to its own pool.

Next, we investigated the CT states among the individual pools of WT and N98H mutant. The CT energies and couplings were obtained from a diabatization procedure devised in ref (25), applied to the excited states of the a603–a609 dimer in its optimized structures. The calculations revealed two CT states of similar energies but with opposite charge separations, which we name a603^+^a609^–^ and a609^+^a603^–^. As there are two local excitations on either a603 or a609, in the following we analyze four different LE–CT couplings. The couplings a603*–(a603^+^a609^–^) and a609*–(a603^–^a609^+^) both involve an electron transfer between virtual orbitals of the two Chls and can be labeled as electron transfer (ET) couplings, whereas the remaining couplings, a609*–(a603^+^a609^–^) and a603*–(a603^–^a609^+^), involve occupied orbitals of the two Chls and will be denoted as hole transfer (HT) couplings.^26^

For all of the WT structures, the lowest CT state is a603^+^a609^–^, whose average energy is very similar among different replicas and pools (Figure 3). Only in the case of MD2, we observe more conformations with low CT energies; however, the average energy is only about 180 cm^–1^ larger than for the other MD1 replica. This is also true for the second CT state (a603^–^a609^+^), and in this case, the difference in the average energy is even lower, around 100 cm^–1^. In the mutant, instead, a603^–^a609^+^ becomes the lowest CT state, even if its energy is still about 1000 cm^–1^ larger than that for the lowest CT state of the WT. A drastic increase in energy is also found for a603^+^a609^–^, which is ∼3000 cm^–1^ higher than that in the WT. Although the a603–a609 pair presents larger distances in the mutant, this difference seems too small to account for the rise in the CT energies. Instead, we can connect this effect to the lack of a H-bond of a609 with Asn98, which could cause a destabilization of the negative charge on a609, raising the energy of a603^+^a609^–^. The LE–CT couplings show more significant differences between the individual pools of structures of the WT and also with the N98H mutant. As suggested by our intermolecular coordinate, in the MD1 replica, pool 1 features smaller couplings than pool 2, and both present smaller couplings than the MD2. For the N98H mutant, all couplings are decreased with respect to the WT-MD2 values, and in some cases they are even lower than MD1-pool1. This striking difference between the WT and the N98H mutant is expected to cause a different effect of the CT state on the exciton states.

**Figure 3:**
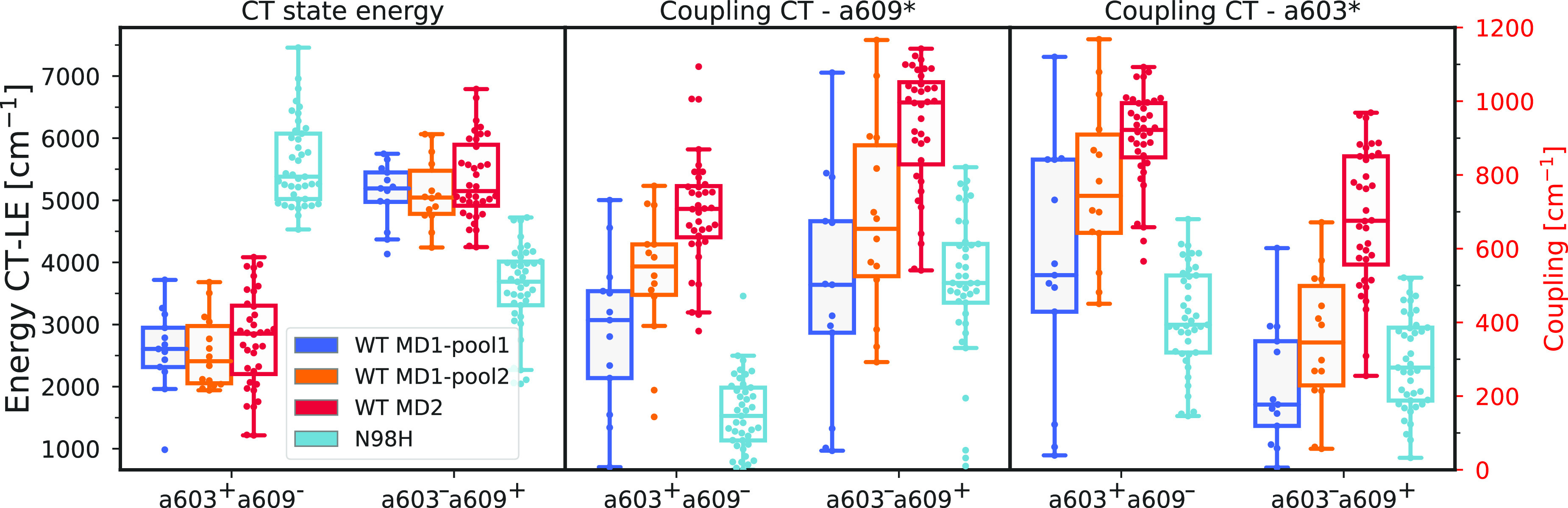
Exciton parameters of the CT state. Comparison of the CT state energies and CT-LE couplings for the individual clusters and replicas of the WT and the N98H mutant. The CT energies are given relative to the LE state. The two lowest CT states are indicated by the character of the CT state. The different colors represent the WT pools defined in Figure 2d and mutant N98H.

As previously suggested,^24^ we observe a good correlation between molecular orbital overlap and the coupling of the CT state to the LE states (Figure S7 in the SI). More specifically, the LUMO–LUMO overlap correlates almost perfectly with the ET couplings, i.e., a603*–(a603^+^a609^–^) and a609*–(a603^–^a609^+^). Instead, the HOMO–HOMO orbital overlap is well correlated with the HT couplings, i.e., a609*–(a603^+^a609^–^) and a603*–(a603^–^609^+^). All CT–LE couplings are therefore very sensitive to the mutual geometrical position of the Chl pair. This explains why even small changes in the mutual sliding of the chlorophylls, as observed in Figure 2, can dramatically influence the couplings.

The energies of the CT states are related to the difference between the HOMO and LUMO energies of the electron donor and acceptor, respectively. However, here the correlation is less clear than for the couplings (Figure S7 in SI). Notably, the HOMO–LUMO energy gap does not show large variations among the WT conformations, whereas it is much larger in N98H. As a consequence, the HOMO and LUMO energies can in part explain the striking difference between the WT and mutant.

Importantly, the excitonic coupling between the LE states remains almost the same in all of the pools considered here (Figure S8 in the SI). In fact, contrary to the couplings with CT states, the exciton couplings do not depend on the orbital overlaps, but rather on the Coulomb interaction between transition densities. This makes the exciton couplings much less sensitive to small changes in the orientations of the Chls.

To investigate how the CT states influence the spectra of Lhca4, we computed the exciton states and the optical spectra, including or excluding the CT state in the exciton system, and artificially tuned the CT energies and couplings. We investigate these effects on WT-MD2 because this pool features the largest CT couplings among all of the studied structures.

As shown in Figure 4a the largest impact of CT states is on the lowest exciton state, formed by the Chl pair a603–a609. This state is lowered by ∼500 cm^–1^, whereas the upper exciton formed by the same pair is almost unaltered, similarly to the other excitons of Lhca4. This shift is present in spite of the very large energy separation of the LE and CT states (thousands of cm^–1^) and can be explained by the strong coupling between the CT and LE states, which is 1 order of magnitude larger than the typical “strong” couplings within LE states. Notably, a similar effect was also observed in the LH2 complex of purple bacteria.^20,25^ The CT coupling results in substantial energy separation between the lowest exciton and upper excitons. Based on the eigenstates of the average Hamiltonian, we estimate that the lowest exciton has ∼12% contribution from the CT states.

**Figure 4:**
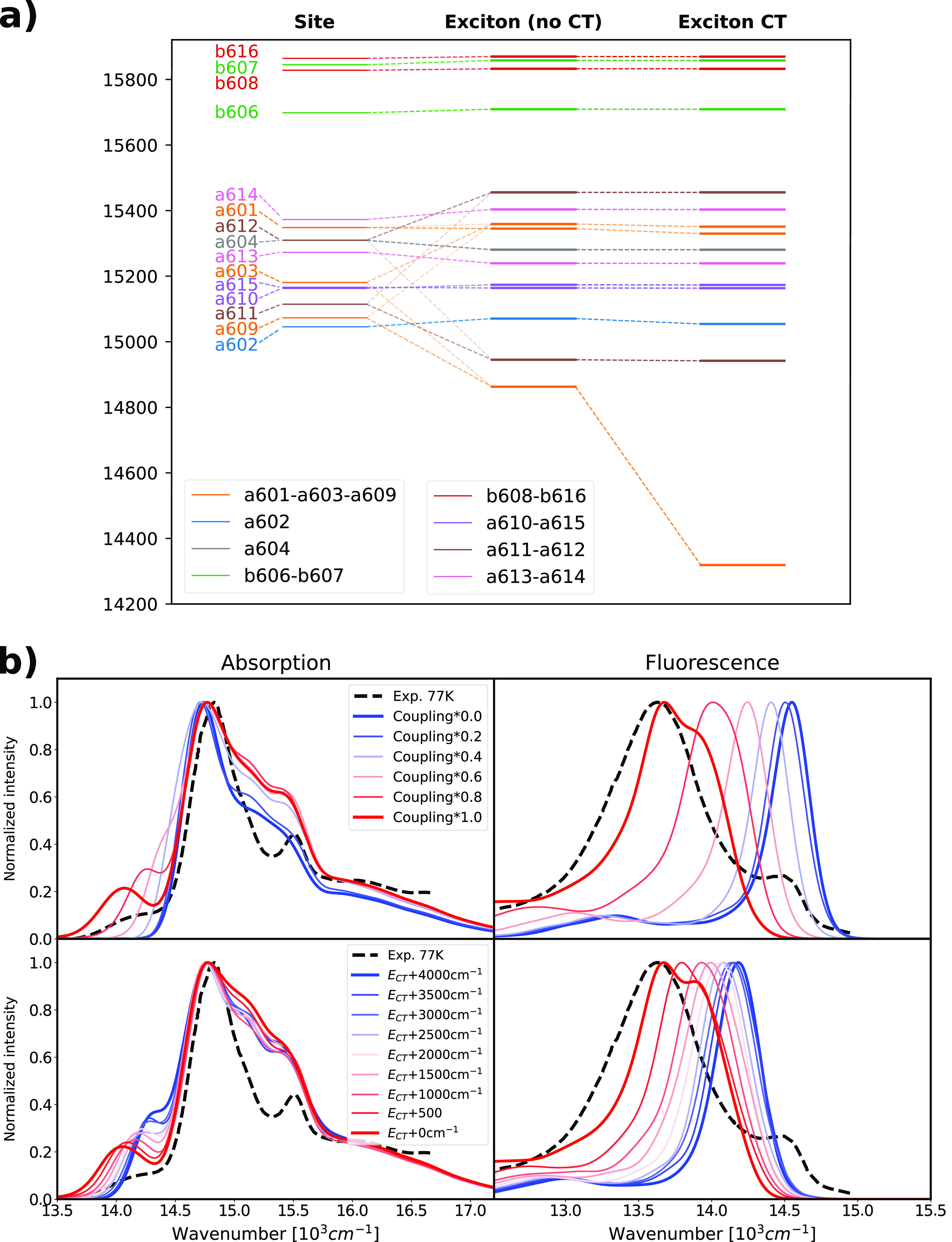
Exciton state composition and effect of the CT states and resulting optical spectra for the structures in WT-MD2. (a) Representation of the contribution of the exciton states and the effect of the CT states. The analysis was performed on the average WT-MD2 Hamiltonian. The leftmost column represents the site energies of the Chl Q_*y*_ states (CT states are not shown); the middle column shows the exciton energies obtained excluding the CT states from the Hamiltonian, while the right column shows the exciton energies obtained from the full exciton-CT Hamiltonian. (b) Effect of the coupling between Q_*y*_ and the CT state and CT state energy on the optical spectra. The coupling was gradually reduced from the MD2 values to zero by applying a uniform scaling, as shown in the legend. The CT state energy was gradually increased from the MD2 values to +4000 cm^–1^, fixing the couplings. All computed spectra were shifted by −1261 cm^–1^ to compare with experiments. Experimental absorption and fluorescence spectra are taken from ref (27) and shown as dashed lines.

To relate these results to the experiments, in Figure 4 we simulated absorption and fluorescence spectra starting from the WT-MD2 structures (see Section S1.2 in the Supporting Information).

Our simulations of absorption spectra (Figure 4b, left panels) predict a small red-shifted band, well separated from the main peak of the spectrum, which clearly corresponds to the CT-mixed lowest exciton state. To understand the impact of CT mixing, we further generated artificial models in which either the LE–CT couplings or the CT energies were modified. We first gradually reduced the couplings to both CT states to zero (Figure 4b, top), i.e., decoupled the CT states from the exciton states. Scaling the CT couplings leads to a blue-shift of the low-energy band, which virtually disappears when CT couplings decrease below 50% of their original values. In the second test, we gradually increased the energy of the CT states by different amounts (Figure 4b, bottom), keeping the couplings fixed. Increasing these energies reduces the CT mixing into the lowest exciton state and also blue shifts the low-energy band. However, in this case the red-shifted band remains visible, even after increasing the CT energy by 4000 cm^–1^. A secondary effect of the strong coupling of the lowest exciton state to the CT state is a redistribution of the reorganization energy. Due to the charge separated character, the CT state has a higher interaction with the environment, which inevitably leads to larger reorganization energies than those typical for the LE states. This redistribution of the reorganization energy leads to a broadening of the line shape corresponding to the lowest exciton and a larger Stokes shift and broadening of the fluorescence spectra (Figure 4b). On the other hand, for MD1-pool1 and the N98H mutant, a smaller effect of the CT state on the exciton system is observed due to the small coupling between CT and LE states (Figure S9 in the SI).

For a proper description of the fluorescence spectra, it is important to consider that different mixing between CT and LE states occurs in the ground- and excited-state geometry (Figure S10). This effect originates from the significantly different energy gap between LE and CT states in the two geometries. To take this factor into account, the emission spectra are described by a different exciton-CT Hamiltonian, where the CT–LE energy difference is reduced by a correction factor obtained from calculations on the dimer (see Section S1.3 in the SI). In the fluorescence spectra (Figure 4b, right panels), we see that by scaling the LE–CT couplings, the fluorescence band drastically blue shifts, consistent with the disappearance of the low-energy absorption band, and reaches ∼14500 cm^–1^ when the LE–CT coupling is zero. Raising the CT energy has a similar effect, but once again, even a 4000 cm^–1^ increase does not decouple the LE and CT states.

These findings suggest that the magnitude of the CT couplings is fundamental for the appearance of low-energy absorption and fluorescence features. Indeed, the CT couplings in WT-MD2 are as large as 1000 cm^–1^ and halving them is sufficient to remove the red-shifted spectral features almost completely. The CT state energies, which are far higher than the exciton energies, seem to mainly affect the exact positions of the red-shifted bands.

We finally compared the optical spectra of the different Lhca4 conformations (MD1-pool1 and pool2 and MD2) and the mutant in Figure 5. In the same figure we also report the experimental spectra of both WT and N98H. Our calculations capture the most important features of the absorption spectra, in particular the weak low-energy band at ∼14000 cm^–1^, in addition to the typical LHC features comprising the Chl a peak at around 14800 cm^–1^ and a small Chl b peak at around 15500 cm^–1^, although they overestimate the intensity around 15000 cm^–1^. Strikingly, among the WT conformations, only MD2 and MD1-pool2 reproduce the low-lying weak band with good accuracy, while this feature is not present in the spectra calculated from MD1-pool1. This is not surprising given the above analysis (Figure 4), as the structures from MD1-pool1 show much smaller CT couplings (Figure 3).

**Figure 5:**
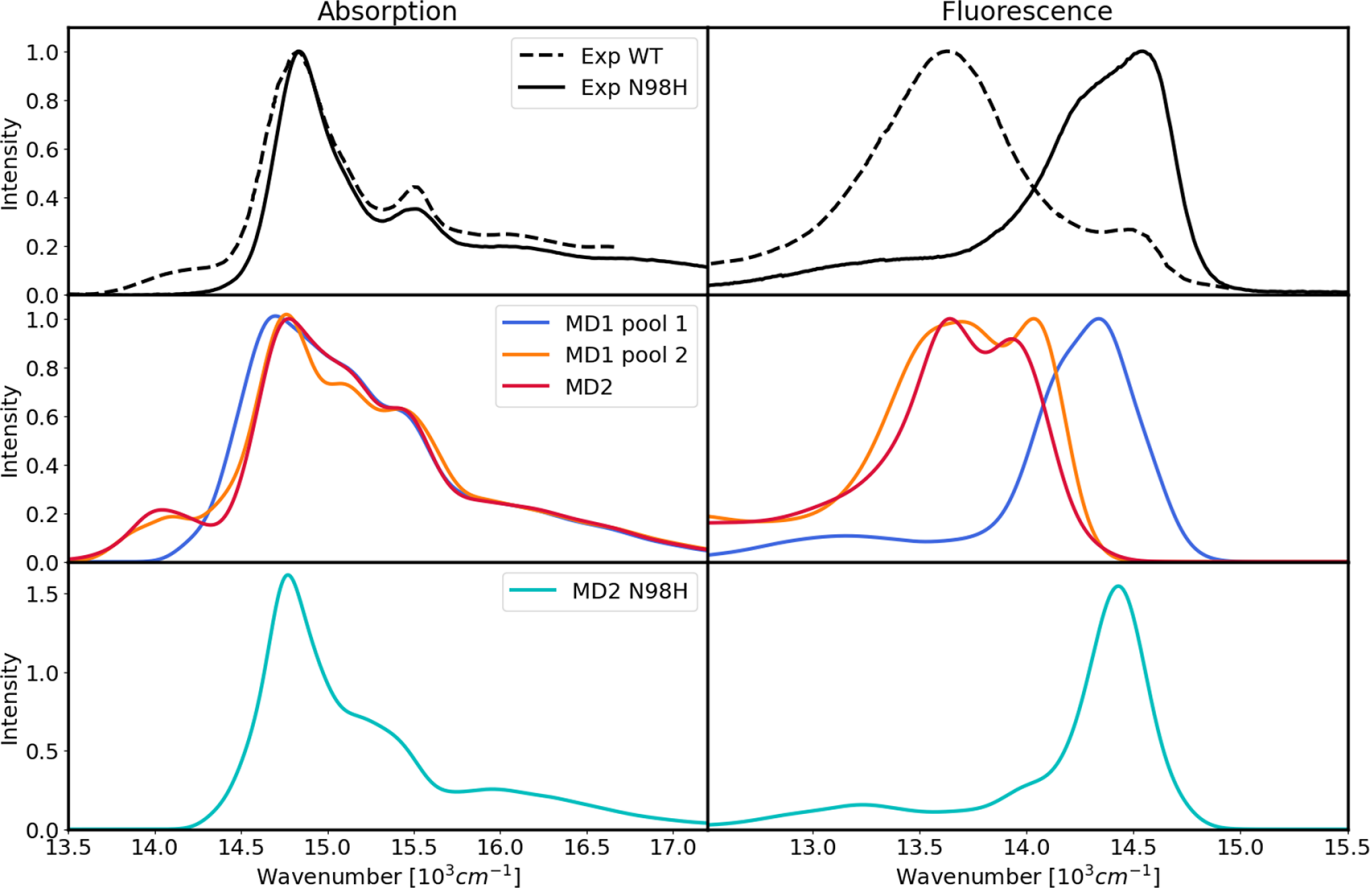
Optical spectra of Lhca4 at 77 K. Top row: experimental absorption (left) and fluorescence (right) spectra of Lhca4. The results for MD4 are shown in Figure S11 in the SI. Middle row: optical spectra of the different replicas and pools of the WT. Bottom row: optical spectra of the N98H mutant. All computed spectra were shifted by −1261 cm^–1^ to compare with experiments. The experimental spectra were obtained from ref (27).

Moving to the fluorescence spectra, we can notice a substantial difference between the conformations. Indeed, the fluorescence of MD1-pool1 is blue-shifted by almost 1000 cm^–1^ with respect to the other WT conformations. Comparing these two cases with the experiment, we see that MD1-pool1 corresponds to the band at ∼14500 cm^–1^, while both MD2 and MD1-pool2 reproduce the band at ∼13500 cm^–1^. Therefore, according to our calculations, the dual fluorescence observed for Lhca4 arises from the presence of multiple conformations of the complex with different levels of LE–CT coupling. Notably, the blue-shifted fluorescence occurs at higher energies than the lowest absorption band at ∼14000 cm^–1^, which would be impossible for a single conformation. The presence of a red-shifted fluorescence indeed correlates with the low-energy absorption shoulder as they are both absent in MD1-pool1 and present in the other two conformations.

These results demonstrate that very similar conformations can give rise to drastically different spectroscopic properties in the WT, both in fluorescence and in absorption. Indeed, a small relative displacement of Chls a603 and a609 (Figure 2a) can make the low-energy band appear or disappear and shift the fluorescence by almost 1000 cm^–1^. This can explain the results of single-molecule experiments, which have shown that Lhca4 can reversibly switch between different states with blue- or red-shifted emission.^12^

To simulate the relative intensities of the two emission peaks, we would need to obtain very accurate estimations for the conformational equilibrium populations in addition to the quantum yields of the emitting states. Therefore, we refrain from computing a single fluorescence spectrum averaging on the conformations. Nonetheless, we can notice that the structures in MD1-pool1 represent a minor fraction of the Lhca4 ensemble, which is consistent with the lower intensity of the blue-shifted emission. This is also the case in single-molecule experiments on Lhca4, which showed a minor but nonzero population of blue-shifted emitting states.^12^ Summarizing, we can identify the emission at ∼14500 cm^–1^ (690 nm) with the structures from MD1-pool1, whereas the red-shifted emission at ∼13500 cm^–1^ (740 nm) can be identified with both MD1-pool2 and MD2 structures.

To simulate the N98H mutant, the Q_*y*_ properties and couplings were taken from MD2 of the WT and only the CT state properties were recomputed from the MD sampling of the mutant. Even with this approximation, we find quite different spectra compared to the WT case and similar to the case without CT–LE couplings (Figure 4b), suggesting that in N98H the CT state has a negligible effect on the exciton states. This is mainly due to the very small coupling of the lowest CT state to the a603 LE state and its higher excitation energy, as shown in Figures 5, 3 and S11 in the SI. The absorption spectrum is more similar to that of MD1-pool1 of the WT, although it is somewhat narrower. Also in the experiments, the main Chl band is broader in the WT than in N98H, which indicates that a smaller coupling to CT states, like in MD1-pool1, still has an impact on the absorption spectrum. The simulated N98H fluorescence band peaks at ∼14500 cm^–1^, which corresponds to the blue-shifted band of the experimental WT fluorescence spectrum. Taken together, these results show virtually no sign of low-lying states, in agreement with the experiments.^13−15,28,29^ Here we have considered only the Asn to His substitution in the axial ligand of a603, but it is worth mentioning that the analogous substitution with Gln has the same effect of removing all low-energy signatures.^27^

In this work, we have shown that the combination of molecular dynamics, multiscale QM/MMPol calculations, and spectral simulations confirms that the low-lying red states and red-shifted fluorescence in Lhca4 originate from the interplay of exciton and CT states within the a603–a609 Chl pair. Revealing the molecular mechanism of formation of the low-lying exciton-CT states is a crucial step in the manipulation of the light harvesting, transfer, and quenching properties of LHCs. In addition, the two identified structural pools in this work could also provide a basis for the structural investigation of the two observed conformations in single-molecule spectroscopy of the Lhca4 complex.^12^ Likewise, the microscopic Hamiltonian calculated in this work represents an independent foundation for simulations of exciton dynamics of Lhca4.^30,31^ Our results can also explain experimental results obtained with the LHCs of Photosystem II (i.e., Lhcb proteins), such as LHCII and CP29. Lhcbs generally do not show evidence of low-lying states in absorption spectra and usually presents 680 nm fluorescence. However, far-red emission at 740 nm was also observed in these complexes in single-molecule spectroscopy and upon aggregation in ensemble experiments.^12,32−34^ What is more, the far-red emission of CP29 has apparently the same characteristics as the Lhca-like emission from PSI-LHCI.^35^ These far-red-emitting states seem common to all LHCs, although they are dominant only in Lhca. The conformations that favor far-red emission are stabilized in Lhcas, whereas they are not populated in Lhcbs under specific conditions. The available evidence points to a similar origin of far-red states in Lhca and Lhcb complexes, suggesting that the mechanism highlighted here for tuning the exciton-CT mixing could also be active in Lhcb complexes.

## Supporting Information Available

Additional theoretical details of the MD simulation, exciton model and optical spectra calculation, including the figures with additional structural information, CT state properties, optical spectra of the WT MD4 structures, scheme of the CT-exciton state mixing, and table with the averaged exciton Hamiltonian for the Lhca4 (PDF)
Transparent Peer Review report available (PDF)
