# Supplementary material for: Origin of Low-Lying Red States in the Lhca4 Light-Harvesting Complex of Photosystem I
Source: J Phys Chem Lett. 2023 Sep 13;14(37):8345–52. doi: 10.1021/acs.jpclett.3c02091 (PMC10518868; doi:10.1021/acs.jpclett.3c02091)
Supplement: Supplementary file 1 — jz3c02091_si_001.pdf [file jz3c02091_si_001.pdf]

Supporting Information: On the Origin of Low-lying Red States in the Lhca4 Light-harvesting Complex of Photosystem I

Vladislav Sláma[1,*], Lorenzo Cupellini[1,*], Vincenzo Mascoli[2], Nicoletta Liguori[2], Roberta Croce[2], and Benedetta Mennucci[1]

[1]Department of Chemistry and Industrial Chemistry, University of Pisa, 56124 Pisa, Italy
[2]Faculty of Science, Vrije Universiteit Amsterdam, 1082 TA Amsterdam, Netherlands
[*]Corresponding Author, e-mail: lorenzo.cupellini@unipi.it (LC), vladislav.slama@dcci.unipi.it (VS)

S1 Exciton model with charge-transfer states

The excitation properties and the optical spectra were modeled with the Frenkel excition model. Within the Frenkel exciton model the excited states of the whole system constructed from the basis of localized states on individual sites. The exciton Hamiltonian is constructed from the excitation energies of individual pigments and coupling between them. The resulting exciton states are constructed from diagonalization of the following Hamiltonian

$$H_{ex} = \sum_i \varepsilon_i |i\rangle \langle i| + \sum_{j\neq i} V_{ij} |i\rangle \langle j| \tag{S1}$$

where ε_i is excitation energy of the pigment i embedded in the protein and $V_{ij} = V_{ij}^{Coul} + V_{ij}^{MMpol}$ is electronic coupling between pigment i and j, including the effects of the polarizable environment.

In order to include the Charge-Transfer (CT) states between the pigment pairs, we have to extend the exciton Hamiltonian of Eq. (S1) with the CT state energies $\varepsilon_k^{(CT)}$ and couplings $V_{ki}^{(CT-LE)}$ between the CT and locally excited (LE) states

$$H = H_{ex} + \sum_k \varepsilon_k^{(CT)} |k\rangle \langle k| + \sum_{ki} V_{ki}^{(CT-LE)} (|k\rangle \langle i| + |i\rangle \langle k|) \tag{S2}$$

where the index i runs over the LE states and k over the CT states. The couplings $V_{ki}^{(CT-LE)}$ are nonzero only between the intermolecular CT state and locally excited state localized on one of the pigments forming the CT state. The excitation energies of the CT state and its couplings to the locally excited states were obtained from FCD diabatization procedure on the basis of the dimer excited states. [1]

The resulting exciton states $|M\rangle$ can be written as a linear combination of locally excited pigments $|i\rangle$:

$$|M\rangle = \sum_i C_i^M |i\rangle \tag{S3}$$

where c_i^M is the coefficient of the local excited state i in the wavefunction of the exciton state M.

S1.1 Site energies

The site energies of the individual pigments were obtained from a two-step procedure, computing excitation energies for pigment in the protein environment and in vacuo to separate environmental and geometrical contribution as in our previous work.[2,3] The large variability of the site energies in the MD ensemble comes from the bond-length variations. These “fast” degrees of freedom are not properly described within the classical force-field, which replaces the true QM forces with simple parametric potential, and they are included in the spectral density. Therefore the bond-length contribution ΔE^{bond} was subtracted from the excitation site energies. The remaining geometrical contribution was averaged over the structures of each pigment. The environmental contribution E^{env} was modeled by normal distribution which parameters were obtained from the environmental contribution computed for each MD structure separately. The final corrected site energy for the pigment i at configuration t and replica r reads as

$$E_{i,t,r} = E_0 + \left\langle \Delta E_{i,t,r}^{\text{bond}} \right\rangle_{i,t,r} + \left\langle \Delta E_{i,t,r}^{\text{distortion}} \right\rangle_{t} + E_{i,t,r}^{\text{env}} \tag{S4}$$

where E_0 is the excitation energy of reference pigment structure.

S1.2 Optical spectra

The optical spectra are simulated using exciton model and the cumulant expansion formalism. This method is widely employed in the modeling of light-harvesting complexes and assumes small off-diagonal exciton vibrational coupling in the exciton basis, which is reasonable assumption for the locally excited states.[4,5] Within this approximation, the absorption $\alpha(\omega)$, fluorescence $I(\omega)$ and circular dichroism $CD(\omega)$ spectra are obtained as a sum over exciton states:[6]

$$\alpha(\omega) \propto \omega \sum_M |\mu_{0M}|^2 D_M(\omega) \tag{S5}$$

$$I(\omega) \propto \omega^2 \sum_M p_M |\mu_{0M}|^2 \tilde{D}_M(\omega) \tag{S6}$$

$$CD(\omega) \propto \omega \sum_M R_M D_M(\omega) \tag{S7}$$

where p_M corresponds to the Boltzmann factor $p_M = \exp[-\varepsilon_M/k_B T]/\sum_N \exp[-\varepsilon_N/k_B T]$ and $\boldsymbol{\mu}_M = \sum_i c_i^M \boldsymbol{\mu}_i$ is the transition dipole between the ground and the M-th exciton state. For the CD spectra the intensity of the individual transition is given by

$$R_M \propto \varepsilon_M \sum_{i>j} c_i^M c_j^M \boldsymbol{R}_{ij} \cdot \boldsymbol{\mu}_i \times \boldsymbol{\mu}_j \tag{S8}$$

The homogeneous lineshape $D_M(\omega)$ and fluorescence lineshape are obtained in the cumulant expansion formalism as:

$$D_M(\omega) = \int_{-\infty}^{\infty} e^{-i(\omega_M - \omega)t - g_M(t)} dt \tag{S9}$$

$$\tilde{D}_M(\omega) = \int_{-\infty}^{\infty} e^{-i(\omega_M - \omega)t + 2i\lambda_M t - g_M^*(t)} dt \tag{S10}$$

where $g_M(t)$ is the lineshape function of exciton M and λ_M is corresponding reorganization energy. Assuming that the individual sites are uncorrelated and neglecting the effect of coupling fluctuations on the homogeneous lineshape, we can write

$$g_M(t) = \sum_i \left|c_i^M\right|^4 g_i(t) \tag{S11}$$

where $g_i(t)$ is the lineshape function of site i, which is obtained from the spectral density $J(\omega)$:

$$g_i(t) = -\int_0^\infty \mathrm{d}\omega \frac{J_i(\omega)}{\pi\omega^2}\left[\coth\left(\frac{\beta\hbar\omega}{2}\right)\big(\cos(\omega t) - 1\big) - i\left(\sin(\omega t) - \omega t\right)\right] \tag{S12}$$

S1.3 Fluorescence line shapes

The strong coupling of the CT state to the environment leads to a structure-dependent energy gap between the LE and CT states. Thus, the mixing with CT states is dramatically different in the ground and excited-state geometry (Figure S10). In order to include this effect quantitatively, we use a different Hamiltonian for the fluorescence and for the absorption ones. For fluorescence spectra we use a Hamiltonian appropriate for the excited-state geometry, modeled by a shift of the CT state excitation energy with respect to the ground-state one. This shift is estimated from the gradient of the CT and LE states at the ground-state geometry of the a603-a609 dimer. The excited-state gradients were projected onto the normal modes to obtain the nuclear displacement and reorganization energies corresponding to the relaxation in the lowest exciton state. We performed this calculation for 10 selected frames and obtained an average shift of the CT energy $\Delta E_{\text{relax}} = -1275$ cm^{-1} compared to the LE states. This shift was applied to the CT energy to obtain the modified Hamiltonian used in fluorescence spectra.

S1.4 Spectral densities

The spectral density of the pigments was modeled by two contributions. The first contribution corresponds to the intramolecular vibrations coupled to the electronic excited states and is modeled by underdamped Brownian oscillator. The vibrational contribution for the chlorophylls was modeled by a sum of overdamped Brownian oscillator and 48 high-frequency modes obtained from the experimental fit of the optical spectra.[4] The second contribution originates from the nuclear motion of the environment and it is modeled by an overdamped Brownian oscillator. For the chlorophylls we used a reorganization energy λ_{env}=60cm^{-1} as previously employed for LHCSR1[3].

The vibrational contribution for the CT states was obtained within the vertical gradient method.[2,7,8] The structure of the dimer was optimized at the ONIOM level with the B3LYP functional where only the chlorophylls were included in the QM layer and allowed to move. At the optimized geometry the normal mode analysis was performed using the same approach as for the geometry optimization. The gradients of the excited and ground state potential surfaces were computed at the TDA-DFT approach with ωB97X-D functional. The total reorganization energy of the intramolecular part of the spectral density is $\lambda_{vib}^{CT} = 2596$ cm^{-1}. Reorganization energy of the environmental contribution was obtained from the fluctuation of the CT state energy for the chlorophyll dimer at the QM optimized geometry. We used the relation

$$\lambda_{env}^{CT} = \frac{\sigma\left(E_{CT}^{QMopt}\right)^2}{2k_BT} \tag{S13}$$

The environmental reorganization energies for the CT states are $\lambda_{\text{env}}^{603^+609^-} = 1337$ cm^{-1} and $\lambda_{\text{env}}^{603^-609^+} = 941$cm^{-1}.

S2 Structure and Molecular Dynamics

S2.1 Structure Preparation

For the MD simulations we started from the Lhca4 crystal strcuture of Mazor at al. (PDB: 5L8R).[9] The crystal structure was cleaned and only DGDG and MGDG (digalactosyl diacyl glycerol and monogalactosyl diacyl glycerol) were kept because they are in the vicinity of the chlorophylls and might have a structural function. The *tleap* module of AmberTools was employed to add hydrogens to prepare the initial structure. All titratable residues were kept in their standard protonation state, except for Chl-binding histidines, which were δ-protonated in order to allow Mg binding and glutamic acid (GLU 145) is protonated to form the hydrogen bond with the nearby chl-b. This protonation was chosen on the basis of pKA calculations with the H++ server[10] at pH 7. After a first *in vacuo* minimization with a 10 kcal mol^{-1} Å^{-2} harmonic restraint on backbone atoms, Lhca4 was embedded in a model DOPC membrane and solvated with water molecules. The AMBER ff14SB force field was used for the protein. Carotenoids were described with the force field by Prandi *et al.*[11], and chlorophyll *a* was described with the force field by Ceccarelli *et al.*[12] with Zhang *et al.* modifications[13]. Lipids were described with the lipid14 force-field[14], and water was described with the TIP3P model. The parameters for nonstandard lipids MGDG and DGDG were obtained based on the lipid-14 parameters where missing parameters were substituted from Amber GAFF forcefield. The atomic charges are computed from simultaneous fitting of the MGDG and DGDG with constraining the charges of the lipid tails to the ones in lipid-14 forcefield. This way we assure compatibility of the fitted forcefield parameters with the lipids of the membrane. The parameters of the β-carotene were derived from the zeaxanthin parameters of the forcefield by Prandi *et al.*[11]. The N98H mutant was obtained by manually replacing the Asn sidechain with a His sidechain. Before proceeding with the all-atom MD simulations, the N98H mutant was relaxed with a 1 μs united-atom MD simulation with the GROMOS force field[15] as detailed in Section S2.3. The rest of the preparation was repeated as detailed above.

S2.2 MD simulations

The MD simulation protocol was based on a previous study on CP29[16]. A first minimization was performed only on the lipids that made close contact with the protein or cofactors. Then, the entire system was minimized without constraints. A 5 ps simulation in the NVT ensemble followed by a 100 ps simulation in the NPT ensemble were used to heat the system to 300 K, with the protein and cofactors constrained by a 10 kcal mol^{-1} Å^{-2} harmonic restraint. The box equilibration step was performed in the NPT ensemble by gradually releasing the restraints to 0.4 kcal mol^{-1} Å^{-2} in 5 ns. An additional 100 ns simulation was performed to equilibrate the loops and other mobile regions of the protein, restraining only the backbone of the transmembrane helices by a 0.4 kcal mol^{-1} Å^{-2} harmonic restraint. The production simulations (replicas MD1–MD4) were performed freely for 1 μs in the anisotropic NPT ensemble. The Langevin thermostat and (for NPT simulations) the Monte Carlo anisotropic barostat were used to control temperature and pressure, respectively. The SHAKE algorithm was used in all simulations along with a 2 fs time step. Particle-mesh Ewald electrostatics with a 10 Å non-bonded cutoff was used.

S2.3 Details on the united-atom simulation

The Lhca4 structure from Mazor *et al.*[9] was prepared using GROMACS and embedded in a solvated membrane. After a first minimization, the system was heated for 10 ps in the NVT ensemble and then equilibrated with four NPT dynamics (10 ns each) with restraints on protein and cofactors, whereas membrane and water molecules were left unbiased. The constraints (10000 kJ mol^{-1} nm^{-1} initially) were gradually reduced step after step (10000 $\rightarrow$ 1000 $\rightarrow$ 500 $\rightarrow$ 200 kJ mol^{-1} nm^{-1}). After this initial relaxation, Asn-98 was manually replaced with His, followed by an additional

10 ns simulation using 200 kJ mol^{-1} nm^{-1} restraints on the protein an cofactors. Finally, 1 μs of unrestrained NPT dynamics was performed. The force field for the cofactors was taken from previous work.[15]

S3 Excited-state calculations

The site energies for the LE states and couplings between them were computed at TD-DFT level with M06-2X functional and 6-31G(d) basis set which we found to describe well the lowest excited states of the light harvesting antennas in our previous works[3,17]. Only the lowest excited state Q_y was included in the exciton system for the chlorophylls. The effects of the protein and the environment were included through a polarizable QM/MM methodology (MMPol)[18–20]: the pigments are treated at TD-DFT level, whereas the rest of the atoms (the protein, membrane and the solvent) were treated at MM level. A radius of 15 Å was used for the polarization cutoff. The MMPol atoms were described using charge and polarizability parameters derived by Wang et al.[21]. In particular, the parameter set based on Thole's linear smeared dipole field tensor was used, in which 1-2 and 1-3 interactions are excluded. A truncated QM model was used for the Chl in which the phytyl chain has been cut and the dangling bond has been saturated with a hydrogen atom. The atoms of the phytyl chain were included as MMPol sites. For computing the CT states within the a603-a609 dimer, the geometry of the system was optimized at the ONIOM level where all the residues within 5 Å from the chlorophyll pair were allowed to move and the chlorophylls with the binding residues (i.e. Asn98/His98 for Chl a603, and Glu153 and Arg156 for Chl a609) were included in the QM layer. The structure optimization was performed with B3LYP DFT functional and 6-31G(d) basis set. The a603-a609 dimer excited states were computed at TDA-DFT level with ωB97XD functional and the same basis set as for the LE states. The ωB97XD functional was used because it recovers 100% HF exchange for large distances. The CT excitation energies and their couplings to the LE states were obtained with the multistate FED-FCD diabatization procedure[1] where 20 excited states of the dimer were included into the calculation. The two lowest CT states $603^{+}609^{-}$ and $603^{-}609^{+}$ were included into the the exciton system of the LE states to simulate the optical spectra. For including the CT states into the exciton system the CT energy was determined by the difference between in excitation energy between CT and LE states calculated from the diabatization which allows us to use the CT states together with LE states obtained with different methods.[22]

The state specific polarization correction for the CT excitation energies[20] was computed for the first two replicas for the optimized structures at the QM level and averaged over 27 and 36 different conformations of the system for MD1 and MD2, respectively. The average state specific correction to the CT excitation energies $\Delta E_{SS}^{CT1} = -1991$ cm^{-1} and $\Delta E_{SS}^{CT2} = -1848$ cm^{-1} was used for the first two CT states of the replica 1 and $\Delta E_{SS}^{CT1} = -1553$ cm^{-1} and $\Delta E_{SS}^{CT2} = -1285$ cm^{-1} for the replica 2. To avoid a costly calculation of the state specific correction to the CT state energies for the mutant, we have used the same values as for the replica 1 of the WT. The TDA-DFT calculation with ωB97X-D overestimates the energy of the CT state. To correct this error we performed ADC(2) gas-phase calculations for 8 randomly chosen conformations and compared the LE-CT energy gap with the ones obtained from TDA-DFT calculation. The comparison yields a correction factor $\Delta E_{ADC(2)}^{CT} = -1315$ cm^{-1} for the method for computing the CT state excitation energies.

S4 Supplementary Figures

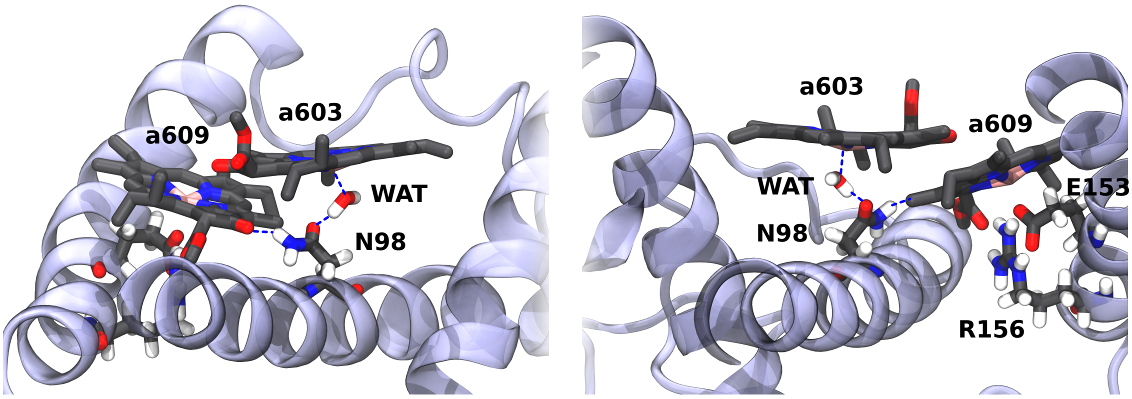

Figure S1: **Structure of the Chl CT pair in the Lhca4 WT MD4.** QM/MM optimized structures of WT MD4 with water molecule entered between Chl a603 and its ligand, Asn98, forming a stable hydrogen bond with the oxygen atom of Asn98 and binding the Mg atom of the chlorophyl. The structure is stable during the 1μs simulation. Blue dashed lines represents hydrogen bonding.

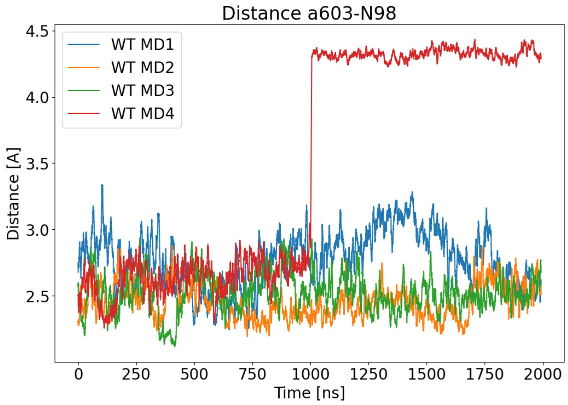

Figure S2: **Evolution of the distance between a603 and N98 binding residue.** For the MD1-3 there is stable binding of the Chl a603, while for the MD4 water molecule entered between Chl a603 and binding residue N98 in around 1 μs and formed stable binding.

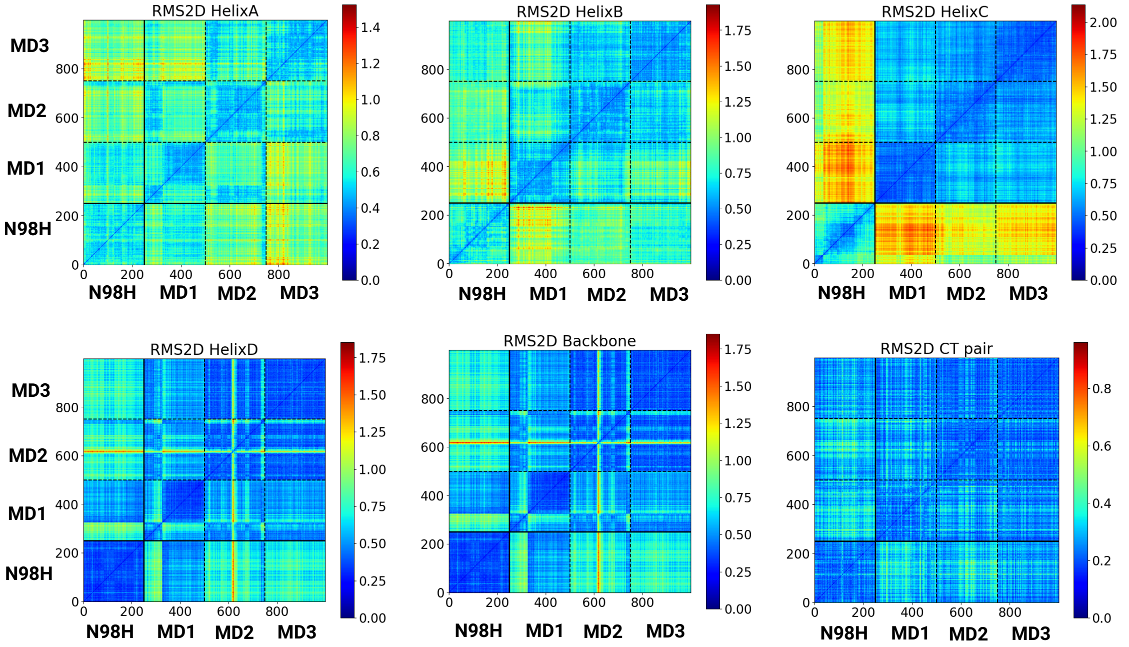

Figure S3: **RMS2D analysis of the MD structures.** The comparison shows the similarity between individual replicas of the WT. Note the different color scale in each plot. The MD2 and MD3 of the WT are more similar with each other than with the MD1. The differences between replicas for helix D are caused by few frames for which the helix D is more rotated in the plane defined by the membrane. The largest differences between the WT and the mutant occur in helix C due to the shift of the helix C towards the stromal part of the membrane in the N98H mutant.

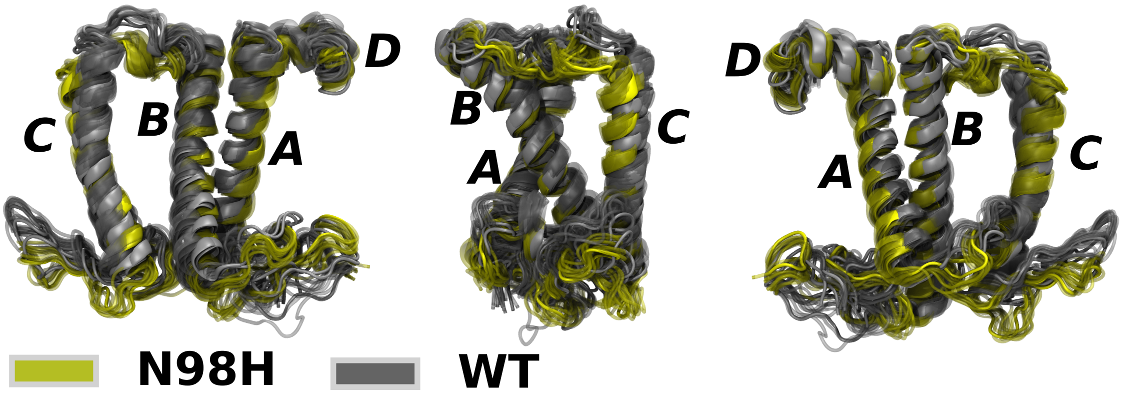

Figure S4: **Comparison of the protein conformations for the WT and N98H mutant.** Three different views on the protein structures and its variation among different replicas and between WT and the mutant. The letters label individual helices of the complex. Both WT and N98H mutant have very similar structures with largest difference in helix C which is shifted towards the stromal part of the membrane and more rotated with respect to the main transmembrane helices A and B.

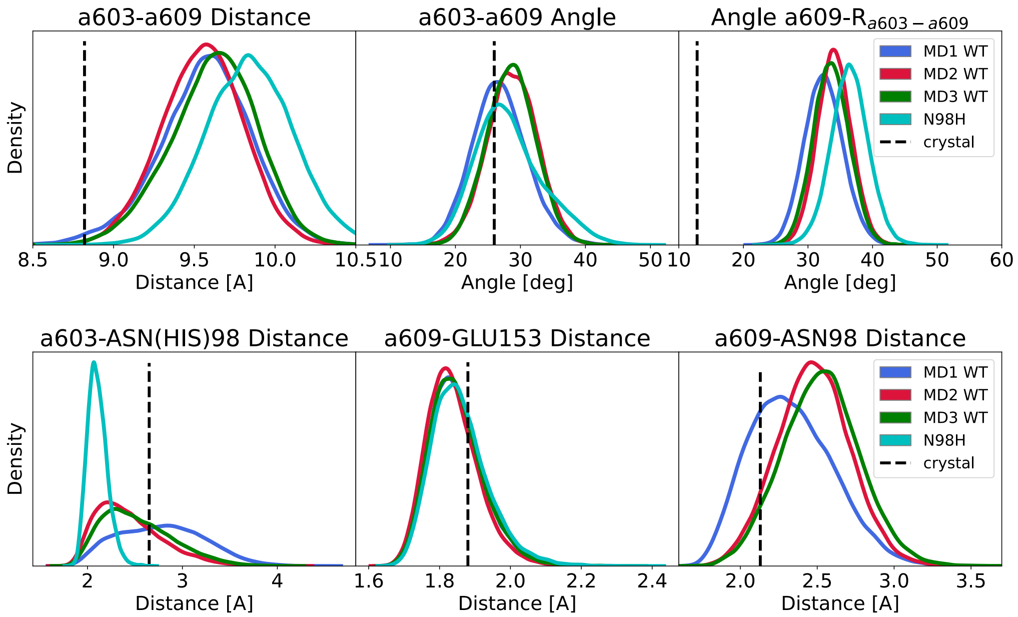

Figure S5: **Structural analysis of the CT pair.** Structural analysis of the a603-a609 dimer for individual replicas of the Lhca4 WT (MD1, MD2 and MD3) and the N98H mutant. The angle a609-R$_{a603-a609}$ corresponds to the angle between the Chl y-axis and the center-center position vector, while the angle a603-a609 refers to the angle between the y-axes of the Chls.

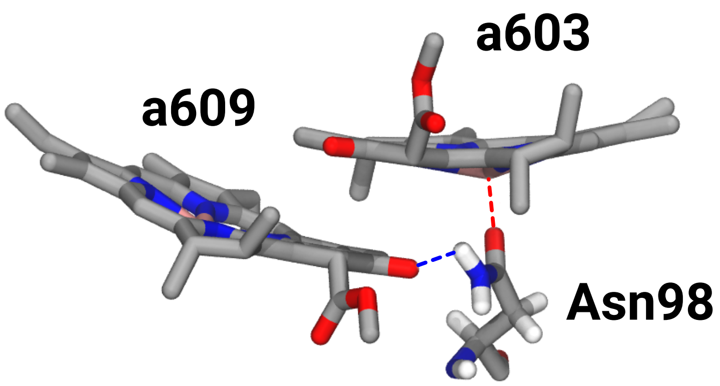

Figure S6: **Structure of the Chl CT pair in the Lhca4 WT.** The mutual conformation of the chlorophyll pair in the WT is stabilized by hydrogen bonding with the asparagine residue Asn98.

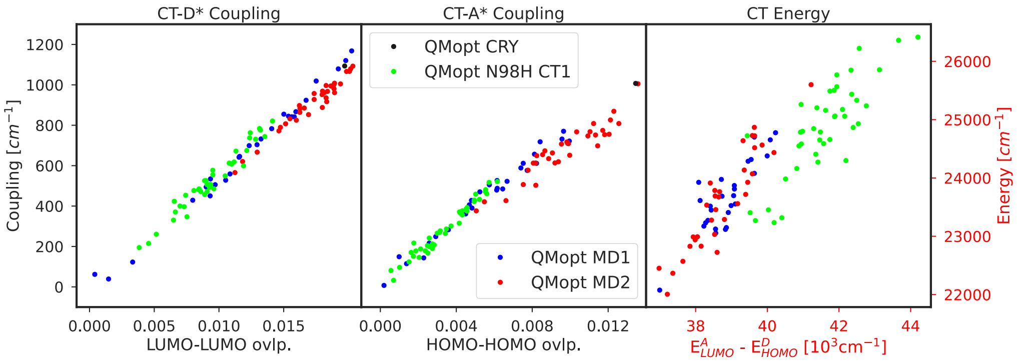

Figure S7: **Relation between CT state properties and single molecular quantities.** Dependence of the CT state properties on the quantities obtained from the single molecule calculation for the lowest CT state. Coupling of the LE and CT state is very well correlated with the molecular orbital overlap. The changes in the CT state excitation energy can be reasonably well explained by the changes in the energy difference between MO energies of the electron donor HOMO orbital and accceptor LUMO orbital. We note that for WT the lowest CT state has $603^{+}609^{-}$ character while for the N98H mutant it has $603^{-}609^{+}$ character.

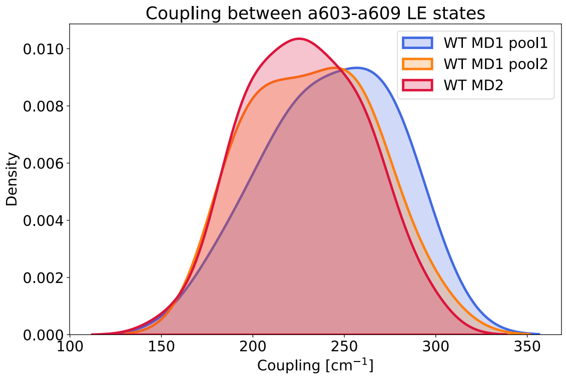

Figure S8: **Exciton coupling between LE states for the CT pair.** Distribution of the exciton couplings between LE states of the CT pair, Chls a603 and a609, for each pool of structures. Even thou the distributions of the couplings are slightly different for each pool of structures, the differences are much smaller than for the the couplings of the LE states to the CT state.

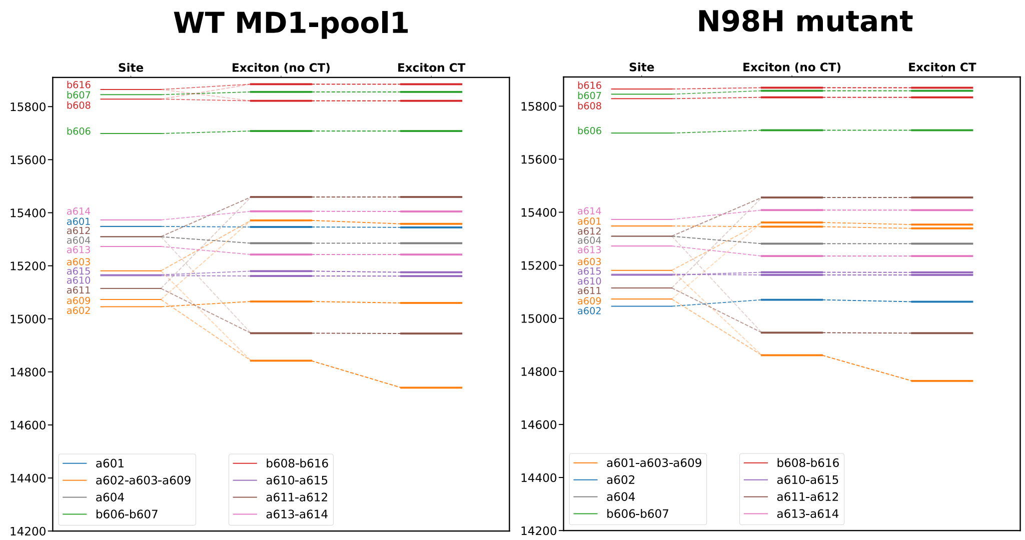

Figure S9: **Exciton state composition and effect of the CT states.** Representation of the contribution of the exciton states and effect of the CT states. The analysis was performed on the averaged Hamiltonian over different structures. The leftmost column represent the site energies of the Chl LE states (CT states are not shown); the middle column shows the exciton energies obtained excluding the CT states from the Hamiltonian, while the right column shows the exciton energies obtained from the full exciton-CT Hamiltonian.

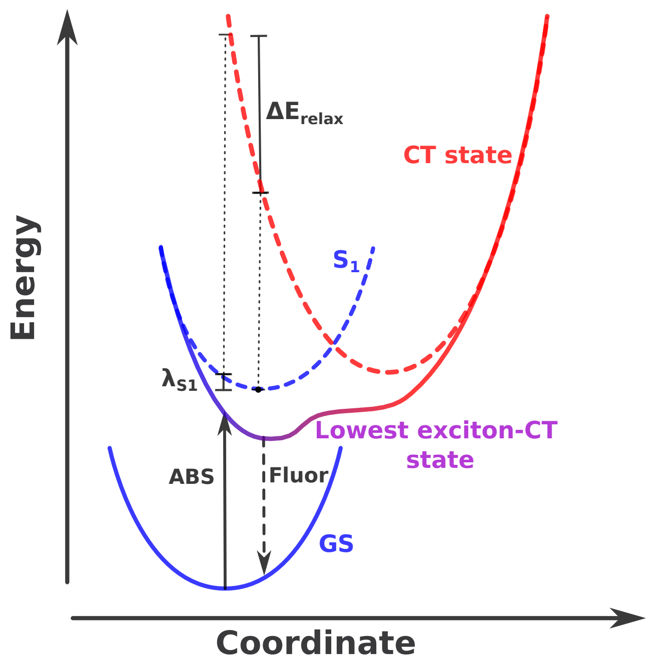

Figure S10: **Scheme of the electronic states and mixing with the CT state.** Schematic representation of the electronic potential surfaces. Color of the lowest exciton-CT state represents contribution of the LE (blue) and CT (red) states to the exciton. The CT state has much larger reorganization energy λ than the LE states. This leads to larger energy gap between LE states and the CT state for the ground state (GS) optimal geometry than for the excited state optimal geometry. The difference in energy gap ΔE_{CT-LE} can be obtained as $\Delta E_{CT-LE} = \lambda_{S_1} - \Delta E_{relax}$. Dependence of the energy gap on the geometrical coordinate leads to coordinate dependent expansion coefficients of the exciton states. This is for the purpose of this work approximated by two different Hamiltonians, one for the absorption and the other for the fluorescence spectra.

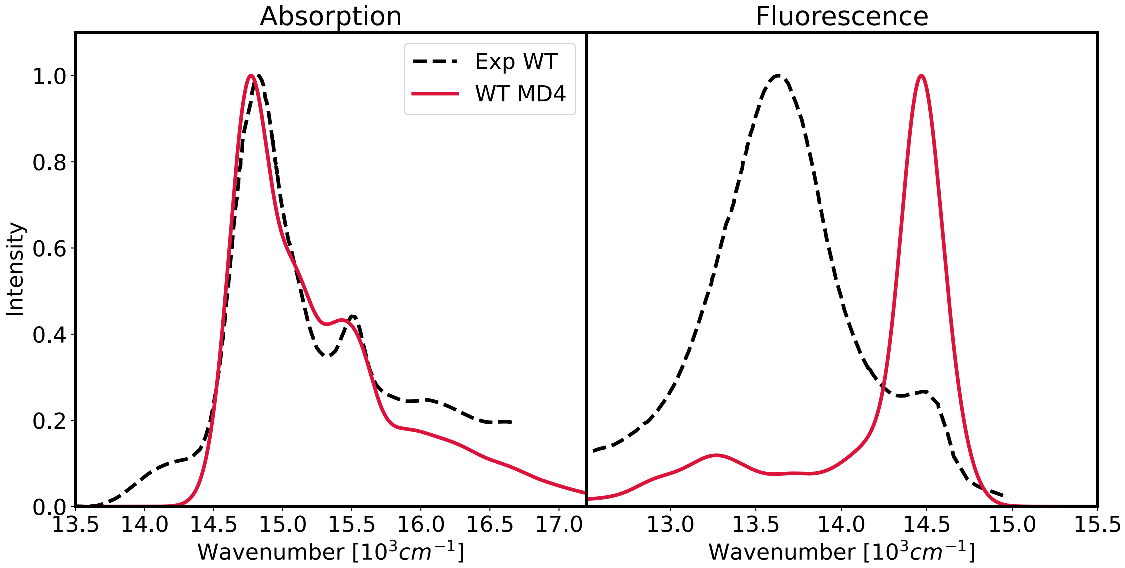

Figure S11: **Optical spectra of the WT MD4.** Absorption and fluorescence spectra of the WT MD4. Only the structures after 1 μs were considered, where Chl a603 is water-ligated (Figure S1). Due to the larger distance between Chl pair a603-a609 there is only very small coupling between CT and LE states, hence no low lying red states are observed in the spectra. The experimental spectra were obtained from ref. 23.

S5 Supplementary Table

Table S1: Exciton Hamiltonian of Lhca4 averaged over the MD. All data in cm^{-1}

	**a601**	**a602**	**a603**	**a604**	**b606**	**b607**	**b608**	**a609**	**a610**	**a611**	**a612**	**a613**	**a614**	**a615**	**b616**
**a601**	16550.2	-24.9	12.0	0.0	0.0	0.0	0.0	0.0	-0.1	-41.4	6.8	2.7	-9.7	0.0	0.0
**a602**	-24.9	16278.7	44.5	9.0	7.7	8.5	-8.1	-44.2	-15.6	-10.9	23.5	-2.4	0.7	-2.3	0.0
**a603**	12.0	44.5	16431.5	-5.4	-10.5	10.6	3.1	228.7	19.4	-1.2	-2.6	4.1	-8.8	8.2	-4.4
**a604**	0.0	9.0	-5.4	16445.4	102.5	34.5	-2.1	-7.5	-3.8	-4.6	3.4	2.0	-6.2	1.7	3.1
**b606**	0.0	7.7	-10.5	102.5	16752.0	35.4	-2.7	-4.5	-2.2	0.0	2.7	2.2	0.0	-2.0	-0.7
**b607**	0.0	8.5	10.6	34.5	35.4	16846.1	-5.5	-19.4	1.8	0.0	3.4	4.3	-3.7	-5.0	-0.2
**b608**	0.0	-8.1	3.1	-2.1	-2.7	-5.5	16886.8	50.1	74.7	7.4	-0.8	0.0	0.0	3.4	20.5
**a609**	0.0	-44.2	228.7	-7.5	-4.5	-19.4	50.1	16444.0	-8.2	6.3	-1.6	-5.5	0.0	-11.9	13.2
**a610**	-0.1	-15.6	19.4	-3.8	-2.2	1.8	74.7	-8.2	16350.5	-42.7	39.0	9.8	-0.3	4.6	7.7
**a611**	-41.4	-10.9	-1.2	-4.6	0.0	0.0	7.4	6.3	-42.7	16425.7	221.5	-10.2	-0.8	0.0	0.0
**a612**	6.8	23.5	-2.6	3.4	2.7	3.4	-0.8	-1.6	39.0	221.5	16549.3	1.9	3.2	0.0	0.0
**a613**	2.7	-2.4	4.1	2.0	2.2	4.3	0.0	-5.5	9.8	-10.2	1.9	16535.4	-61.1	0.0	0.0
**a614**	-9.7	0.7	-8.8	-6.2	0.0	-3.7	0.0	0.0	-0.3	-0.8	3.2	-61.1	16557.1	0.0	0.0
**a615**	0.0	-2.3	8.2	1.7	-2.0	-5.0	3.4	-11.9	4.6	0.0	0.0	0.0	0.0	16516.2	2.0
**b616**	0.0	0.0	-4.4	3.1	-0.7	-0.2	20.5	13.2	7.7	0.0	0.0	0.0	0.0	2.0	16794.9

References

[1] Nottoli, M. *et al.* The role of charge-transfer states in the spectral tuning of antenna complexes of purple bacteria. *Photosynthesis Research* **137**, 215–226 (2018).

[2] Cignoni, E., Slama, V., Cupellini, L. & Mennucci, B. The atomistic modeling of light-harvesting complexes from the physical models to the computational protocol. *J. Chem. Phys.* **156**, 120901 (2022).

[3] Prandi, I. G., Sláma, V., Pecorilla, C., Cupellini, L. & Mennucci, B. Structure of the stress-related LHCSR1 complex determined by an integrated computational strategy. *Commun. biol.* **145**, 145 (2022).

[4] Novoderezhkin, V. I., Palacios, M. A., van Amerongen, H. & van Grondelle, R. Energy-transfer dynamics in the LHCII complex of higher plants: modified redfield approach. *J. Phys. Chem. B* **108**, 10363–10375 (2004).

[5] Novoderezhkin, V. I. *et al.* Mixing of Exciton and Charge-transfer States in Light-harvesting Complex Lhca4. *Phys. Chem. Chem. Phys.* **18**, 19368–19377 (2016).

[6] Renger, T. & Müh, F. Understanding Photosynthetic Light-Harvesting: a Bottom Up Theoretical Approach. *Phys. Chem. Chem. Phys.* **15**, 3348 (2013).

[7] Ferrer, F. J. A. & Santoro, F. Comparison of vertical and adiabatic harmonic approaches for the calculation of the vibrational structure of electronic spectra. *Phys. Chem. Chem. Phys.* **14**, 13549–13563 (2012).

[8] Lee, M. K., Huo, P. & Coker, D. F. Semiclassical Path Integral Dynamics: Photosynthetic Energy Transfer with Realistic Environment Interactions. *Annu. Rev. Phys. Chem.* **67**, 639–668 (2016).

[9] Mazor, Y., Borovikova, A., Caspy, I. & Nelson, N. Structure of the plant photosystem I supercomplex at 2.6 å resolution. *Nature Plants* **3**, 215–226 (2017).

[10] Anandakrishnan, R., Aguilar, B. & Onufriev, A. V. H++ 3.0: automating pK prediction and the preparation of biomolecular structures for atomistic molecular modeling and simulations. *Nucleic Acids Res.* **40**, W537–W541 (2012).

[11] Prandi, I. G., Viani, L., Andreussi, O. & Mennucci, B. Combining classical molecular dynamics and quantum mechanical methods for the description of electronic excitations: The case of carotenoids. *J. Comput. Chem.* **37**, 981–991 (2016).

[12] Ceccarelli, M., Procacci, P. & Marchi, M. An ab initio force field for the cofactors of bacterial photosynthesis. *J. Comput. Chem.* **24**, 129–42 (2003).

[13] Zhang, L., Silva, D. A., Yan, Y. & Huang, X. Force field development for cofactors in the photosystem II. *Journal of Computational Chemistry* **33**, 1969–1980 (2012).

[14] Dickson, C. J. *et al.* Lipid14: The amber lipid force field. *Journal of Chemical Theory and Computation* **10**, 865–879 (2014).

[15] de Jong, D. H. *et al.* Atomistic and coarse grain topologies for the cofactors associated with the photosystem II core complex. *J. Phys. Chem. B* **119**, 7791–7803 (2015).

[16] Lapillo, M., Cignoni, E., Cupellini, L. & Mennucci, B. The energy transfer model of nonphotochemical quenching: Lessons from the minor cp29 antenna complex of plants. *Biochimica et Biophysica Acta (BBA) - Bioenergetics* **1861**, 148282 (2020).

[17] Sláma, V., Cupellini, L. & Mennucci, B. Exciton properties and optical spectra of light harvesting complex II from a fully atomistic description. *Phys. Chem. Chem. Phys.* **22**, 16783–16795 (2020).

[18] Curutchet, C. *et al.* Electronic Energy Transfer in Condensed Phase Studied by a Polarizable QM/MM Model. *Journal of Chemical Theory and Computation* **5**, 1838–1848 (2009).

[19] Lipparini, F. General Linear Scaling Implementation of Polarizable Embedding Schemes. *J. Chem. Theory Comput.* **15**, 4312–4317 (2019).

[20] Bondanza, M., Nottoli, M., Cupellini, L., Lipparini, F. & Mennucci, B. Polarizable embedding QM/MM: the future gold standard for complex (bio)systems? *Phys. Chem. Chem. Phys.* **22**, 14433–14448 (2020).

[21] Wang, J. *et al.* Development of Polarizable Models for Molecular Mechanical Calculations I: Parameterization of Atomic Polarizability. *The Journal of Physical Chemistry B* **115**, 3091–3099 (2011).

[22] Cupellini, L. *et al.* Coupling to Charge Transfer States is the Key to Modulate the Optical Bands for Efficient Light Harvesting in Purple Bacteria. *J. Phys. Chem. Lett.* **9**, 6892–6899 (2018).

[23] Wientjes, E., Roest, G. & Croce, R. From red to blue to far-red in Lhca4: How does the protein modulate the spectral properties of the pigments. *Biochim. Biophys. Acta (BBA) - Bioenergetics* **1817**, 711–717 (2012).
