# Supplementary material for: Origin of Low-Lying Red States in the Lhca4 Light-Harvesting Complex of Photosystem I
Source: J Phys Chem Lett. 2023 Sep 13;14(37):8345–52. doi: 10.1021/acs.jpclett.3c02091 (PMC10518868; doi:10.1021/acs.jpclett.3c02091)
Supplement: Supplementary file 2 — jz3c02091_si_002.pdf [file jz3c02091_si_002.pdf]

jz-2023-02091c.R1

Name: Peer Review Information for "On the Origin of Low-Lying Red States in the Lhca4 Light-Harvesting Complex of Photosystem I"

First Round of Reviewer Comments

Reviewer: 1

Comments to the Author

The authors present an elegant and comprehensive combination of molecular dynamics, multiscale quantum chemical calculations, and spectral simulations with the aim to understand the molecular mechanism for formation and fine-tuning of exciton and charge-transfer (CT) interactions in Lhca4. Interestingly, the authors show how extremely sensitive is the coupling between exciton and CT states to very small changes in the relative orientation between the two excitonically coupled a603-a609 chlorophyll pair. By unravelling the precise mechanism of exciton-CT mixing and its consequences on the energy landscape and spectral signatures, the authors provide unvaluable information on how to control the energy transfer dynamics and quenching processes in light-harvesting complexes, which in turn, has far-reach implications in the development of new materials for solar-energy conversion (for instance for photovoltaic applications).

The manuscript is very well-written, the methodology is properly and clearly presented and the conclusions are adequately supported by the data. In addition, the manuscript presents significant new physical insights which will be appealing for a broad physical chemistry audience, not only for specialists working in photosynthesis research.

Therefore, I strongly recommend the manuscript for publication in The Journal of Physical Chemistry Letters after minor revision (my comments are indicated below).

Specific comments:

1- Based on bond distances from MDs, it is suggested that Chl is bound weaklier to Asn with respect to His. I would suggest some care when discussing bonding from molecular mechanics simulations. Can the authors comment on the force field parameters that describe the Chl - Asn and Chl - His bonds?

2- A hydrogen bond between the Asn98 amide group and the carbonyl group of Chl a609 is observed in the WT. In the N98H mutant, this interaction is not present, "as the His N-H group does not have the correct orientation to make the H-bond". However, the structure for the mutant was obtained by manually swapping the sidechain. Is it possible that, if another His rotamer was chosen, this H-bond could be established?

3- Figure S11 contains important information about the mechanism of exciton-CT coupling. I suggest to include a more detailed discussion about this figure in the main text (at some point in page 16).

4- In page 16 (line 29-31), the authors mention “Revealing the molecular mechanism of formation of the low-lying exciton states” should not be “low-lying exciton-CT states”? Similarly, in FigS11 the text on the figure “Lowest exciton state” should not be “Lowest exciton-CT state”?

Minor comments on the text:

Page6 (line 9-10): indicate the a603-a609 distance values.

Page6 (line 49): indicate Fig1c.

Comments on the figures:

Fig1: The legend should indicate the meaning of the dashed lines as indicated in FigS1 “Blue dashed lines represent hydrogen bonding”. In addition, it will be good to indicate the ligation to a609 (in panel c) and to a603 and a609 (in panel d).

FigS5: The colors used are too similar and difficult to distinguish. I suggest to use colors with more contrast among each other and to remove the solid color under the curves, as well as to indicate the meaning of the dashed lines.

FigS7: The legend is missing the reference number. This figure seems to have a wrong numbering, in the mean text it is mentioned after FigS10. The sentence “where Chl a603 water-ligated” should not be “with Chl a603 water-ligated” or “where Chl a603 is water-ligated”?

FigS8: The last sentence in the legend seems unfinished. The colors used are too similar and difficult to distinguish. I suggest to use colors with more contrast among each other.

Minor grammar corrections and typos:

Page3 (line 29-32): The order of this sentence seems wrong "These states exhibit broad bandwidths, even at 4K, and exceptionally red-shifted emission extending the absorption of the complex to the far

red." Perhaps it should read: "These states exhibit broad bandwidths, even at 4K, extending the absorption of the complex to the far red, and yielding exceptionally red-shifted emission."

Page10 (line 45-46): It reads "Notably, the HOMO-LUMO energy gap does to show large variations (...)" It probably means: "Notably, the HOMO-LUMO energy gap does not show large variations (...)"

Page S3: The sentence that starts with "the structure of the dimer was optimized at (...)" is missing a capital T.

FigS3: “repolicas” should be “replicas”

Author's Response to Peer Review Comments:

Reviewer: 1

The authors present an elegant and comprehensive combination of molecular dynamics, multiscale quantum chemical calculations, and spectral simulations with the aim to understand the molecular mechanism for formation and fine-tuning of exciton and charge-transfer (CT) interactions in Lhca4. Interestingly, the authors show how extremely sensitive is the coupling between exciton and CT states to very small changes in the relative orientation between the two excitonically coupled a603-a609 chlorophyll pair. By unravelling the precise mechanism of exciton-CT mixing and its consequences on the energy landscape and spectral signatures, the authors provide unvaluable information on how to control the energy transfer dynamics and quenching processes in light-harvesting complexes, which in turn, has far-reach implications in the development of new materials for solar-energy conversion (for instance for photovoltaic applications).

The manuscript is very well-written, the methodology is properly and clearly presented and the conclusions are adequately supported by the data. In addition, the manuscript presents significant new physical insights which will be appealing for a broad physical chemistry audience, not only for specialists working in photosynthesis research.
Therefore, I strongly recommend the manuscript for publication in The Journal of Physical Chemistry Letters after minor revision (my comments are indicated below).

Authors' Reply: we sincerely thank the Reviewer for their positive assessment of our work.

Specific comments:

1- Based on bond distances from MDs, it is suggested that Chl is bound weaklier to Asn with respect to His. I would suggest some care when discussing bonding from molecular mechanics simulations. Can the authors comment on the force field parameters that describe the Chl - Asn and Chl - His bonds?

Authors' Reply: We agree with the Reviewer that the Chl-ligand interactions, including Mg coordination, are force-field dependent. The Chl-ligand interactions are all described with the nonbonded terms of the Amber force field, that is, point-charge electrostatics and van der Waals (vdW) nonelectrostatic terms. Electrostatic and vdW parameters of Asn and His were taken from the well tested Amber ff14SB protein force field. For the Chl, we have used QM-derived parameters by Ceccarelli et al. with modifications by Zhang et al. as described in the SI. This ff was successfully applied to a number of natural light harvesting systems (Balevičius *et al.*, *Sci Rep.* 2017, **7**, 13956; Prandi *et al. Commun Biol.* (2022), **5**, 145; Bourne-Worster *et al.*, *PNAS* 2023, **120**, e2210811120 and others), however, the distances between Chl and a binding residue were never specifically investigated.

The only direct difference between His-binding and Asn-binding is represented by the different parameters of His and Asn in the protein force field. Although we agree with the Reviewer that some care is needed in interpreting the Mg coordination from MD, the difference observed in our simulations between the two binding residues should be at least qualitatively correct.

2- A hydrogen bond between the Asn98 amide group and the carbonyl group of Chl a609 is observed in the WT. In the N98H mutant, this interaction is not present, "as the His N-H group does not have the correct orientation to make the H-bond". However, the structure for the mutant was obtained by manually swapping the sidechain. Is it possible that, if another His rotamer was chosen, this H-bond could be established?

Authors' Reply: We thank the Reviewer for this comment. The orientation of His98 was determined not only by the chosen rotamer but by relaxation through united-atom MD simulations, whose details are reported in the Supporting Information.

To check what happens when another rotamer is chosen, we performed a short MD simulation after flipping the His98 sidechain. As shown in the Figure below, this different rotamer does not have a favorable orientation of the NH group to form a H-bond with Chl a609. As the His(Nδ-H) direction is almost opposite to the direction of His(Nε)-Mg coordination, such a H-bond would require a very different arrangement of the two Chls. Instead, in the short MD simulation we observed a H-bond with the backbone amide oxygen.

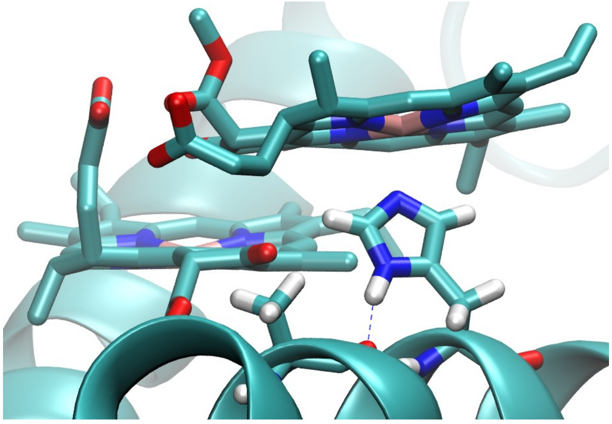

3- Figure S11 contains important information about the mechanism of exciton-CT coupling. I suggest to include a more detailed discussion about this figure in the main text (at some point in page 16).

Authors' Reply: We thank the Reviewer for this suggestion. Indeed Figure S11 was made to explain the method for calculating emission line shapes, which is detailed in the SI section S1.3. To explain this point, we have added the following sentence when first introducing the fluorescence spectra

"For a proper description of the fluorescence spectra, it is important to consider that different mixing between CT and LE states occurs in the ground-state and excited-state geometry (Figure S10). This effect originates from the significantly different energy gap between LE and CT states in the two geometries. To take this factor into account, the emission spectra are described by a different exciton-CT Hamiltonian, where the CT-LE energy difference is reduced by a correction factor obtained from calculations on the dimer (See Section S1.3 in the SI)."

Note that Figure S11 was renamed to Figure S10 due to reordering.

4- In page 16 (line 29-31), the authors mention "Revealing the molecular mechanism of formation of the low-lying exciton states" should not be "low-lying exciton-CT states"? Similarly, in FigS11 the text on the figure "Lowest exciton state" should not be "Lowest exciton-CT state"?

Authors' Reply: We understand that the "exciton" term might be confusing, as usually exciton states are assumed to not include CT mixing. Within our model, any state (and especially the lowest one) can be considered as "exciton-CT". To avoid confusion, we renamed the "lowest exciton" states to "lowest exciton-CT" states where applicable, as suggested by the Reviewer .

Minor comments on the text:

Page6 (line 9-10): indicate the a603-a609 distance values.

Authors' Reply: We thank the reviewer for the suggestion. We included the absolute values of center-to-center distances for the CT pair into the main text.

Page6 (line 49): indicate Fig1c.

Authors' Reply: We have added a reference to Figure 1c when mentioning the hydrogen bond to the carbonyl.

Comments on the figures:

Fig1: The legend should indicate the meaning of the dashed lines as indicated in FigS1 "Blue dashed lines represent hydrogen bonding". In addition, it will be good to indicate the ligation to a609 (in panel c) and to a603 and a609 (in panel d).

Authors' Reply: We thank the Reviewer for the suggestion. The image was changed to include the ligation. The dashed lines are now explained in the figure caption.

FigS5: The colors used are too similar and difficult to distinguish. I suggest to use colors with more contrast among each other and to remove the solid color under the curves, as well as to indicate the meaning of the dashed lines.

Authors' Reply: We agree with the Reviewer that the colored area below the curve leads to a too cluttered figure due to the overlapping curves. We removed the solid color under the curves and made the lines thicker to make it easier to read.

FigS7: The legend is missing the reference number. This figure seems to have a wrong numbering, in the mean text it is mentioned after FigS10. The sentence "where Chl a603 water-ligated" should not be "with Chl a603 water-ligated" or "where Chl a603 is water-ligated"?

Authors' Reply: We thank the reviewer for pointing out the typo in the reference and the caption. The experimental reference and the figure caption was corrected.

We also reordered the SI figures so that former Figure S7 is now Figure S11, and former Figures S8-S11 are now Figures S7-S10. Now the supporting figures are mentioned in order in the main text.

FigS8: The last sentence in the legend seems unfinished. The colors used are too similar and difficult to distinguish. I suggest to use colors with more contrast among each other.

Authors' Reply: We thank the reviewer for the suggestion and for noticing the errors in the caption. In the original version of this Figure, we used the same coloring scheme as in the main text. However, we agree that changing the colors improves the readability, therefore we changed the color scheme to a more contrasted one. We also fixed the last sentence in the caption, which now reads:

"We note that for WT the lowest CT state has $603^{+}609^{-}$ character while for the N98H mutant it has $603^{-}609^{+}$ character."

Minor grammar corrections and typos:

Page3 (line 29-32): The order of this sentence seems wrong "These states exhibit broad bandwidths, even at 4K, and exceptionally red-shifted emission extending the absorption of the complex to the far red." Perhaps it should read: "These states exhibit broad bandwidths, even at 4K, extending the absorption of the complex to the far red, and yielding exceptionally red-shifted emission."

Authors' Reply: We agree with the reviewer that the proposed sentence is more clear and understandable. We corrected the sentence in the main text.

Page10 (line 45-46): It reads "Notably, the HOMO-LUMO energy gap does to show large variations (...)" It probably means: "Notably, the HOMO-LUMO energy gap does not show large variations (…)"

Authors' Reply: We thank the reviewer for pointing out this typo. The sentence was corrected in the main text.

Page S3: The sentence that starts with "the structure of the dimer was optimized at (...)" is missing a capital T.

Authors' Reply: The typo was corrected in the main text.

FigS3: "repolicas" should be "replicas"

Authors' Reply: We thank the reviewer for noticing the typo. The figure caption was corrected.
